# Exploring Deactivation Reasons of Biomass-Based Phosphorus-Doped Carbon as a Metal-Free Catalyst in the Catalytic Dehydroaromatization of *n*-Heptane

**DOI:** 10.3390/molecules29061288

**Published:** 2024-03-14

**Authors:** Fei Yu, Siyuan Liu, Bo Liu

**Affiliations:** 1 Green Chemical Engineering Research Center, Shanghai Advanced Research Institute, Chinese Academy of Sciences, Shanghai 201210, China; yufei@sari.ac.cn (F.Y.); liubo@hongbaoli.com (B.L.); 2 University of Chinese Academy of Sciences, Beijing 100049, China

**Keywords:** metal-free phosphorus-doped, biomass carbon catalysts, dehydroaromatization, deactivation reasons

## Abstract

Catalytic dehydroaromatization of *n*-alkanes into high-value aromatics has garnered extensive interest from both academia and industry. Our group has previously reported that phosphorus-doped carbon materials exhibit high selectivity for C-H bond activation in the dehydroaromatization of *n*-hexane. In this study, using *n*-heptane as a probe, we synthesized biomass-based phosphorus-doped carbon catalysts to investigate the impact of hydrogen heat treatment and carbon deposition on catalyst structure. Despite achieving an initial conversion of *n*-heptane at approximately 99.6%, with a toluene selectivity of 87.9%, the catalyst activity fell quickly. Moreover, longer hydrogen treatment time and higher hydrogen concentrations were found to accelerate catalyst deactivation. Thermogravimetric analysis (TGA) and N_2_ adsorption measurements (BET) indicated that a small amount of coke deposition was not the primary cause of catalyst deactivation. Temperature-programmed desorption of ammonia gas (NH_3_-TPD) revealed a significant decrease in acid-active functional groups. X-ray photoelectron spectroscopy (XPS) and solid-state ^31^P NMR spectroscopy confirmed the reduction of active central phosphorus species. These results suggest that catalyst deactivation primarily arises from the decrease in acidity and the partial reduction of phosphorus-containing groups, leading to a substantial loss of active sites. This work contributes new perspectives to understanding the properties and design improvements of metal-free carbon catalysts.

## 1. Introduction

Aromatics, particularly benzene, toluene, and xylene (BTX), are essential compounds in the chemical industry, serving as fundamental building blocks for various products such as adhesives, rubber, plastics, and other raw materials [1,2,3]. Additionally, they play an important role in enhancing the octane number of petroleum products [4]. However, due to the shortage of traditional fossil resources, the supply of BTX derived from catalytic reforming and catalytic cracking processes fails to meet the escalating market demand [4,5]. Consequently, the conversion of alkanes into aromatics has attracted considerable attention owing to the abundant and low-cost sources of alkanes [5,6]. Although ZSM-5 zeolites have been widely used as commercial aromatization catalysts, the undesired side reactions involving C–C bond cleavage and recombination often result in mixed aromatics as products [7,8]. Therefore, the urgent issue in dehydrogenation transformation is to explore efficient catalysts with remarkable activity, high selectivity, and outstanding stability.

In recent years, various high-performance aromatization catalysts have been developed [9,10]. For instance, Zhou et al. synthesized the Pt/KZSM-5 (deAl) catalyst through the ion exchange method, achieving aromatics selectivity of up to 75.4% and *n*-heptane conversion of 96.1% [11]. Additionally, noble metal Pt-loaded alkaline KL zeolite catalysts have shown efficacy in promoting the aromatization of C_6_~C_9_ alkanes [12,13]. Li et al. employed atomic layer deposition to fabricate a series of metal-modified Pt/KL zeolite catalysts, incorporating metals such as Ba, Zn, Fe, and Co [14,15,16,17]. Notably, the 6Pt/BaKL catalyst demonstrated a remarkable conversion rate of 97% and a toluene selectivity of 92% in the dehydroaromatization of *n*-heptane [14]. Recently, our group has successfully developed a novel method for achieving highly selective dehydroaromatization of *n*-alkanes into aromatic compounds and hydrogen gas using phosphorus-doped activated carbon (P@AC) as a metal-free catalyst [18]. In our previous work, a nearly complete conversion of *n*-hexane into benzene was achieved, with a benzene yield exceeding 95% [19].

Despite the efforts that have yielded promising results, maintaining catalyst stability remains a prevalent struggle during the conversion of light hydrocarbons to aromatics, owing to the high-temperature conditions required for C–H bond activation [20,21]. Catalyst deactivation predominantly arises from carbon deposition due to side reactions, sintering of metal nanoparticles, and poisoning of active sites [22,23]. HZSM-5 zeolite catalysts require frequent regeneration to preserve their well-defined pore structure due to substantial coke from secondary reactions [4,24]. Pt/KL zeolite catalysts often deactivate resulting from the agglomeration of Pt and coke deposition over prolonged operation [25,26]. Similarly, rapid deactivation of P@C during the catalytic conversion of *n*-hexane to aromatics has been observed, with elemental phosphorus identified at the reactor tube’s outlet. However, the causes underlying the deactivation of metal-free phosphorus-doped carbon materials in light hydrocarbon aromatization reactions remain unclear. Early studies have reported that thermal treatments of carbon materials under different atmospheres such as N_2_ and H_2_ can significantly impact their surface chemical properties [27]. Notably, Kundu et al. found that reducing agents like H_2_ have been shown to diminish the thermal stability of phosphorus-containing groups and induce the evolution of functional groups [28]. Inspired by these literatures, our study aims to investigate the effect of hydrogen on the lifetime and activity of P@C catalysts during aromatization.

In this work, we focus on investigating the deactivation reasons of phosphorus-doped biomass carbon catalysts (P@C) during *n*-heptane dehydrogenation aromatization. Specifically, we aim to conduct activity evaluations and structural characterizations of the catalysts subjected to various durations of hydrogen thermal treatment to elucidate the underlying reasons for deactivation. Furthermore, conclusions were validated under different hydrogen concentration conditions. Ultimately, the findings suggest that the loss of catalytic activity may be attributed to the reduction of acid sites and the transformation of phosphorus species.

## 2. Results

### 2.1. Catalytic Performance of P@C

**Figure 1:**
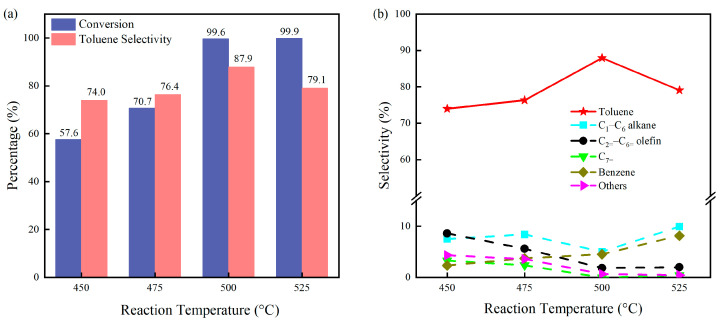
Catalytic activity (**a**) and the products’ selectivity (**b**) of P@C catalyst for *n*-heptane aromatization at different reaction temperatures. Reaction conditions: catalyst loading of 1.0 g, N_2_ carrier gas flow rate of 40 mL/min, and an *n*-heptane model feed flow rate of 1.0 mL/h. Note: The data in the figures correspond to the analysis results obtained at the initial 15 min of the reaction.

Figure 1 presents the performance evaluation of the P@C catalyst in *n*-heptane dehydroaromatization at various reaction temperatures (450 °C, 475 °C, 500 °C, and 525 °C) under atmospheric pressure, using a fixed-bed reactor with a WHSV of 0.68 h^−1^. With increasing reaction temperature, the conversion of *n*-heptane rises, accompanied by an initial increase and subsequent decrease in toluene selectivity. This trend arises from the exothermic nature of *n*-heptane aromatization, resulting in enhanced conversion rates at higher temperatures. However, elevated temperatures promote secondary reactions such as hydrogenation and thermal cracking of *n*-heptane, leading to increased formation of by-products and subsequent reduction in toluene selectivity. Thus, excessively high reaction temperatures impede the dehydroaromatization of *n*-heptane, with 500 °C identified as the optimal temperature for toluene production, evidenced by significantly higher toluene yields compared to other temperatures. Additionally, Figure 1b depicts the product distribution at different reaction temperatures. Despite minor fluctuations in toluene selectivity under different temperature conditions, toluene remains the predominant aromatic product, highlighting the exceptional regioselectivity of the P@C catalyst in *n*-heptane dehydroaromatization.

**Figure 2:**
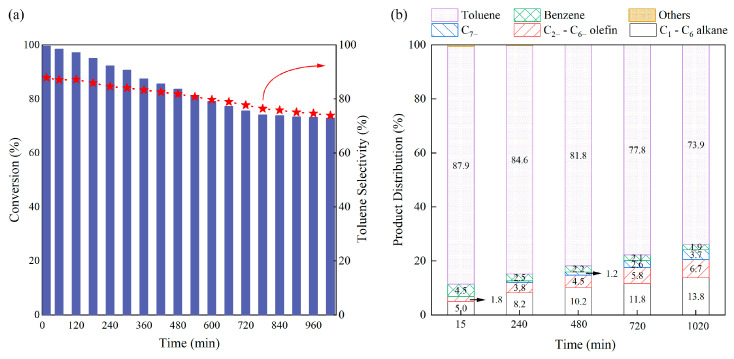
The performance of the P@C catalyst in *n*-heptane dehydroaromatization: (**a**) *n*-heptane conversion and toluene selectivity; (**b**) product distribution at different reaction times. Reaction conditions: 500 °C, catalyst loading of 1.0 g, N_2_ carrier gas flow rate of 40 mL/min, and an *n*-heptane model feed flow rate of 1.0 mL/h. Note: Data for product-selectivity below 1.0% are not labeled in figures. The red arrow of the clock in (**a**) refers to toluene selectivity, and the blue bar chart refers to conversion.

Subsequently, durability testing of P@C was evaluated at a reaction temperature of 500 °C. The results, as shown in Figure 2, indicate that after 15 min of reaction, *n*-heptane conversion reached approximately 99.6%, with a toluene selectivity of about 87.9%. In addition to 4.5% benzene, the hydrocarbon products contain only 5.0% alkenes (C_1_–C_6_ alkane), and 1.8% alkanes (C_2=_–C_6=_ olefin), attributing to the thermal cracking side reactions of *n*-heptane under high-temperature conditions. Table 1 provides a compilation of various catalysts employed in *n*-heptane aromatization over the past five years. The metal-free P@C catalyst not only boasts low cost but also exhibits high initial catalytic activity and toluene selectivity. However, with prolonged reaction time, the catalyst’s activity declines rapidly. When the reaction time reached 1020 min, the conversion rate of n-heptane catalyzed by P@C had decreased to 73.0%, with a toluene selectivity of approximately 73.9%. As illustrated in Figure 2b, the selectivity towards toluene and benzene gradually decreases, while that towards short-chain alkanes and olefins increases, indicating a gradual decline in the aromatization capability of P@C.

### 2.2. Heat Treatment in H_2_ Atmosphere

#### 2.2.1. Catalytic Performance of P@C-H_2_-x

**Figure 3:**
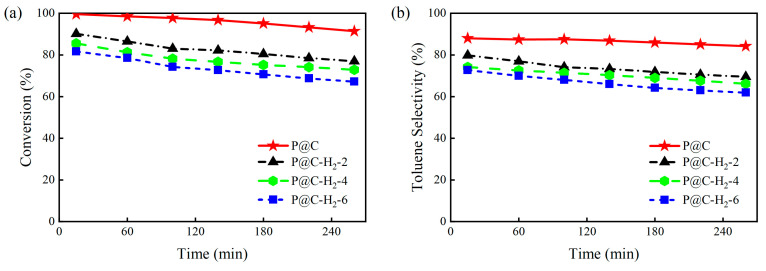
(**a**) *n*-Heptane conversion and (**b**) toluene selectivity of P@C after different H_2_ treatment time. Reaction conditions: 500 °C, catalyst loading of 1.0 g, N_2_ carrier gas flow rate of 40 mL/min, and an *n*-heptane model feed flow rate of 1.0 mL/h. Note: The initial data points were obtained 15 min after the start of the reaction.

The evaluation of *n*-heptane dehydroaromatization catalyzed by P@C-H_2_-x (x = 0, 2, 4, 6) is presented in Figure 3. With the prolonged thermal treatment time of the P@C catalyst in an H_2_ atmosphere, the corresponding initial *n*-heptane conversion for P@C-H_2_-x (x = 0, 2, 4, 6) gradually decreases from 99.6% to 90.1%, 85.5%, and 83.7%, respectively. Similarly, the initial toluene selectivity sequentially decreases to 78.8%, 74.2%, and 72.7%. Figure S3 and Table S1 illustrate the product distribution of each catalyst at 15 min and 260 min of reaction. The results indicate a notable rise in the selectivity towards short-chain low-carbon alkanes (C_1_–C_6_ alkane) for P@C-H_2_-x (x = 0, 2, 4, 6), increasing from 5.0% to 12.0%, 15.0%, and even 18.0%. Moreover, the selectivity towards heptene (C_7=_) gradually increases, while the increase in short-chain olefins (C_2=_−C_6=_ olefin) is less pronounced. These observations confirm that the activity and aromatization performance of P@C are significantly diminished after undergoing thermal reduction treatment with H_2_.

#### 2.2.2. Characterization of P@C-H_2_-x

**Figure 4:**
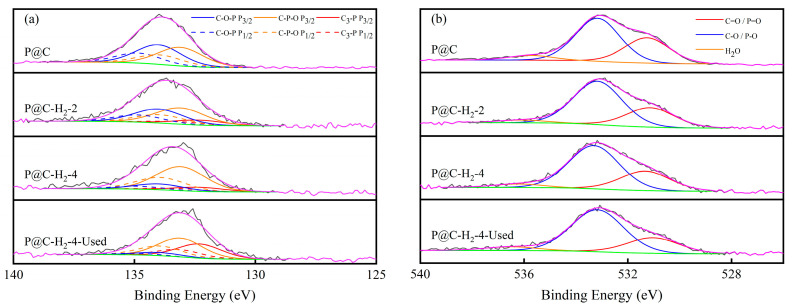
XPS analysis of (**a**) P 2p spectra and (**b**) O 1s spectrum for different P@C-H_2_-x.

To investigate the cause of P@C deactivation induced by H_2_, XPS analysis was performed on P@C-H_2_-x (x = 0, 2, 4, 6), as shown in Figure 4 and Table 2. The P 2p spectrum fitting (Figure 4a) was divided into C–O–P (134.0 eV), C–P–O (133.1 eV), and C_3_-P (131.8 eV) species [32,33]. The O 1s peak spectra (Figure 4b) reveal three distinctive peaks: one at 531.3 eV and another at 533.2 eV, corresponding to double-bonded oxygen (C=O/P=O) and single-bonded oxygen (C–O/P–O) groups, respectively [34]. Additionally, a third peak at approximately 535.7 eV is attributed to adsorbed water [35]. The atomic content of O and P in P@C is 10.17% and 2.31%, respectively. After H_2_ treatment, these values decrease to 5.84% and 1.13% for P@C-H_2_-2, and further decline to 5.40% and 0.82% for P@C-H_2_-4. Analysis of phosphorus species’ evolution suggests that with prolonged H_2_ treatment, there is a noticeable decrease in the relative content of C–O–P, which decreases from 48.7% to 39.8% and 12.2%, while the relative content of C_3_-P exhibits an increasing trend. It is observed that the percentage of C–O/P–O in P@C-H_2_-x tends to increase, rising from 58.8% to 66.2% and 65.4%, whereas the relative content of C=O/P=O gradually declines. Moreover, based on the XPS results of P@C-H_2_-4 before and after the reaction, although the phosphorus content remains consistent, there is a notable increase in the relative content of C_3_-P in P@C-H_2_-4-Used, accompanied by decreases in both C–O–P and C–P–O. These findings suggest that the phosphorus-oxygen groups in P@C undergo reduction by H_2_, making C–O–P more susceptible to transforming to C_3_-P and gradually dissipating from the catalyst surface as elemental phosphorus [36]. Combining the trends in catalyst activity with the evolution of phosphorus species, we propose that phosphorus oxygen functional groups with the C–O–P structure are more likely to be the active sites of the catalyst, which is consistent with our previous studies on active sites [19]. The results indicate that the reduction of phosphorus species may be a primary factor contributing to catalyst deactivation.

**Figure 5:**
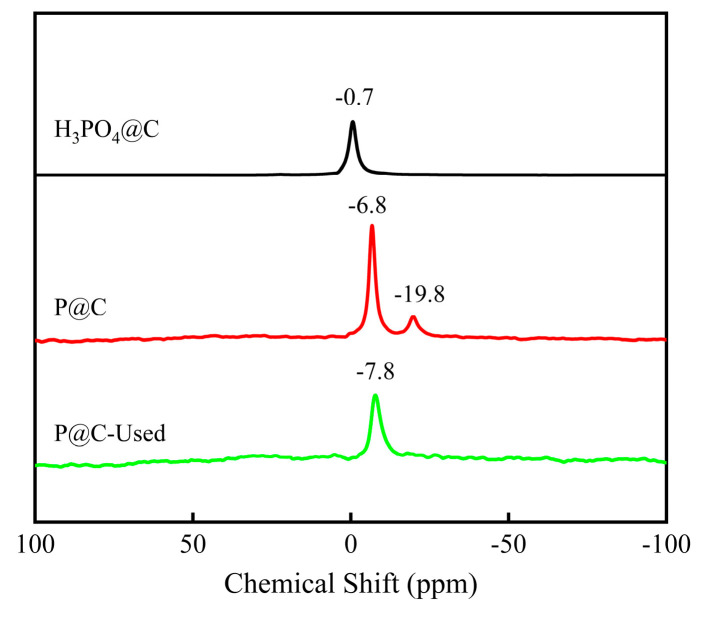
Solid-State ^31^P NMR Analysis of H_3_PO_4_@C, P@C, and P@C-Used catalysts.

^31^P NMR has been used to analyze phosphorus-containing groups in different carbon materials, as shown in Figure 5. Upon the physical adsorption of phosphoric acid onto the precursor starch carbon surface (H_3_PO_4_@C), a peak is observed at −0.7 ppm, near 0 ppm, suggesting the absence of chemical bonding between phosphoric acid and the carbon surface [37]. In P@C, two prominent peaks are discerned at −6.8 ppm and −19.8 ppm, corresponding to C–PO_3_/C–O–PO_3_ and polyphosphoric acid species, respectively [38]. Conversely, in P@C-Used, only a solitary peak emerges at −7.8 ppm, exhibiting a higher field compared to the −6.8 ppm peak, indicative of an enhanced P and C interaction post-reaction, consistent with XPS analysis. The absence of the −19.8 ppm peak implies a reduced presence or depletion of polyphosphoric acid species on the surface following the reaction [39].

**Figure 6:**
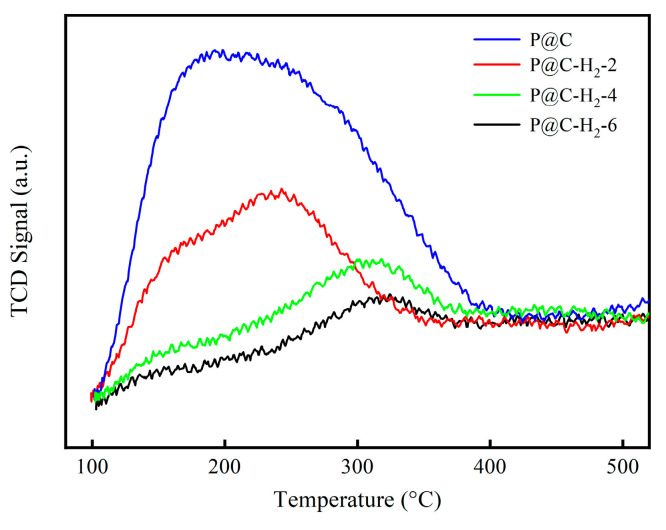
NH_3_-TPD curves of P@C-H_2_-x (x = 0, 2, 4, 6).

The NH_3_-TPD characterization of P@C-x-H_2_ (x = 0, 2, 4, 6) is illustrated in Figure 6 and summarized in Table 3. Initially, the acidity of P@C measured 137.60 μmol/g, while that of P@C-H_2_-2 experienced a rapid decline to 59.48 μmol/g. In contrast, the acidity of both P@C-H_2_-4 and P@C-H_2_-6 remained relatively stable. This observation suggests that certain acidic functional groups of the catalyst undergo gradual reduction upon H_2_ treatment, resulting in decreased acidity. Additionally, BET analysis reveals that H_2_ treatment does not significantly impact the specific surface area, pore volume, and pore size of the catalyst.

Further NH_3_-TPD and BET characterization analyses were conducted on the P@C-H_2_-used sample (x = 0, 2, 4, 6), and the results are presented in Figures S4 and S5, and Table S2. NH_3_-TPD reveals that the acidity of the deactivated catalysts decreases to 20–30 μmol/g, indicating a significant loss of acidic active components after the reaction, with acidic functionality being the main factor contributing to deactivation. These characterization results indicate that after a certain reaction time, the acidity of the P@C catalyst decreases significantly, once again confirming that the deactivation of the catalyst includes the loss of acidity.

#### 2.2.3. Verification in Different H_2_ Concentrations

**Figure 7:**
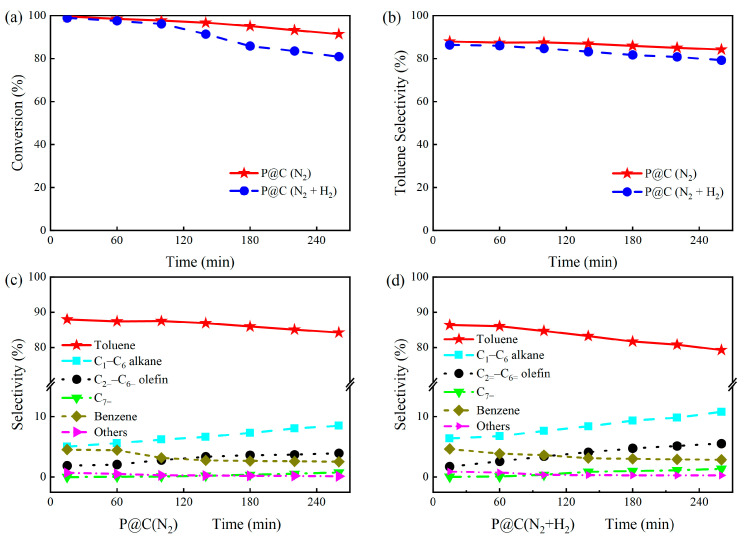
(**a**) *n*-Heptane conversion; (**b**) toluene selectivity; (**c**) the products selectivity of P@C(N_2_) and (**d**) the products selectivity of P@C(N_2_ + H_2_). Reaction conditions: 500 °C, catalyst loading of 1.0 g, and an *n*-heptane model feed flow rate of 1.0 mL/h. Note: P@C(N_2_) denotes the use of pure N_2_ (40 mL/min) as the carrier gas during reaction evaluation, while P@C(N_2_ + H_2_) indicates the use of mixed gas (30 mL/min N_2_ and 10 mL/min H_2_) as the carrier gas.

Through the aforementioned studies, we discovered that H_2_ not only can reduce the phosphorus-containing species but also decrease the acidity of P@C, which are the main reasons for catalyst deactivation. We further explored the catalytic activity and product distribution differences of P@C under different H_2_ concentrations. As shown in Figure 7a,b, compared to P@C(N_2_), during the first 100 min of the reaction, the *n*-heptane conversion and the toluene selectivity of P@C(N_2_ + H_2_) slightly decreased. These values continued to decrease as the reaction time extended, with a higher decrease rate observed in the later stages than in the earlier stages. In contrast, the comparison shown in Figure 7c,d demonstrates that the growth rates of short-chain alkanes (C_1_–C_6_ alkane), olefins (C_2=_–C_6=_ olefin), and heptane (C_7=_) are higher for P@C(N_2_ + H_2_). These results further confirm that the prolonged introduction of H_2_ inhibits the catalytic activity of P@C and accelerates the deactivation process.

Table 4 and Figures S6 and S7 present the acidity and textural properties of the catalysts after the reaction under different H_2_ concentrations. The acidity of P@C(N_2_)-Used and P@C(N_2_ + H_2_)-Used decreased from 137.60 μmol/g to 29.56 μmol/g and 28.54 μmol/g, respectively, while the specific surface area decreased to 946.3 m^2^/g and 1263.9 m^2^/g, respectively. This indicates a slight reduction in the specific surface area and pore volume of P@C(N_2_ + H_2_)-Used. It is hypothesized that the increased hydrogen concentration not only diminishes the catalyst’s aromatization activity but also inhibits coke formation.

### 2.3. Coke Analysis

**Figure 8:**
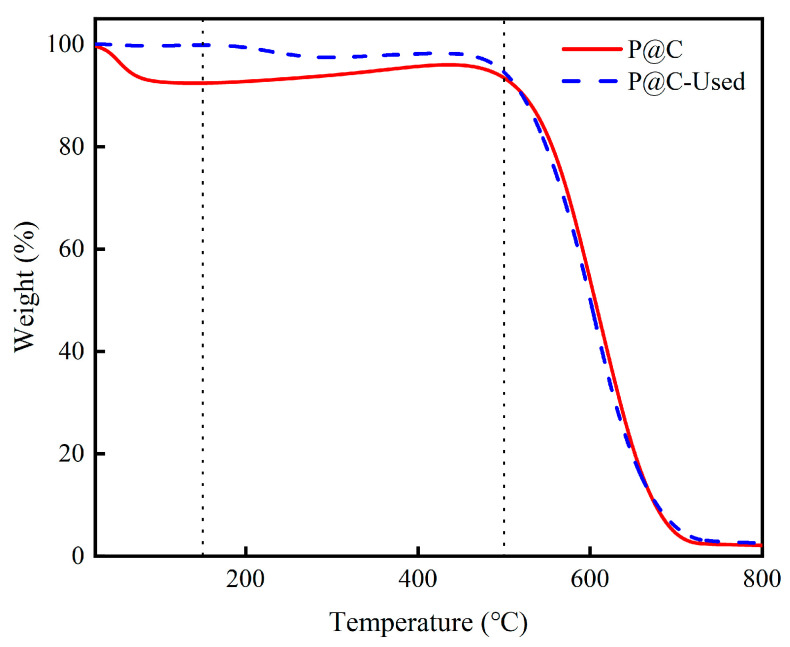
Thermogravimetric analysis results of P@C and P@C-Used in air. Note: The dotted lines are 150 °C and 500 °C respectively.

Coke deposition on the various catalysts was also examined by thermogravimetric analysis (TGA). Figure 8 illustrates the weight loss of the P@C catalyst in air before and after the reaction. The marginal weight reduction observed below 150 °C is attributed to the desorption of adsorbed water, while the significant weight loss in the temperature range of 150–500 °C corresponds to the combustion of carbonaceous species on the catalyst surface. This weight loss in the 150–500 °C range serves as a measure of the deposited coke on the catalyst surface [40,41]. After 260 min of reaction, the coke content on the P@C catalyst was determined to be 6.25 wt%, indicating a remarkably low carbon deposition rate of only 1.20 × 10^−3^ molCarbon/gCat./h. Despite the small amount of carbon deposition, the specific surface areas and pore volumes of P@C exhibit varying degrees of decrease. These carbon deposits may cover active sites, thereby reducing catalyst activity. Table 3 and Table 4 respectively present the carbon deposition rates and textural properties of each catalyst. The carbon deposition rates of P@C-H_2_-x (x = 2, 4, 6) decrease sequentially, being 1.07 × 10^−3^, 0.60 × 10^−3^, and 0.30 × 10^−3^, respectively. The observed decrease in carbon deposition rate may be attributed to the inhibitory effect of the reducing agent H_2_. Considering the changes in carbon deposition quantity and acidity, it is apparent that the latter undergoes more significant changes. Hence, it is conjectured that the deactivation of P@C is likely not predominantly caused by coke formation during the reaction.

## 3. Materials and Methods

### 3.1. Catalyst Preparation

**Figure 9:**
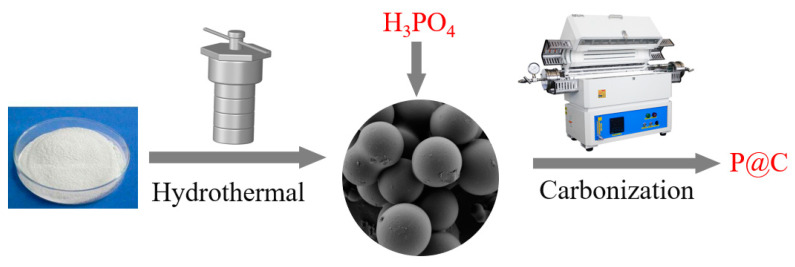
Schematic illustration of P@C synthesis.

All chemicals, including Phosphoric Acid (H_3_PO_4_, 85 wt%, AR) and Soluble Starch ((C_6_H_10_O_5_)_n_, AR), were purchased from General-Reagent (Shanghai, China). A schematic illustration of P@C synthesis is shown in Figure 9. The preparation method for the phosphorus-doped carbon catalyst primarily involves hydrothermal treatment to obtain the carbon precursor, followed by post-treatment for phosphorus doping. Initially, a solution containing 15.0 g of Soluble Starch and 85 mL of deionized water underwent hydrothermal treatment in a Teflon-lined stainless-steel autoclave at 190 °C for 12 h with agitation at 10 rpm. The resulting solid was filtered, washed, and dried overnight at 80 °C to obtain carbon precursors. Subsequently, phosphorus doping was performed by adding a mixture of 10 mL of H_3_PO_4_ solution and 40 mL of deionized water to 6.0 g of carbon precursors, followed by overnight impregnation at 85 °C. The dried samples were then loaded into a quartz tube reactor and carbonized under an N_2_ flow at 800 °C for 5 h, with a ramping rate of 3 °C/min and N_2_ flow rate of 100 mL/min, yielding the phosphorus-doped carbon samples designated as P@C.

The procedure for hydrogen atmosphere heat treatment of the catalyst is detailed as follows: the P@C catalyst was packed into the quartz reaction tube of a fixed-bed reactor and purged with N_2_ at room temperature for 30 min at a flow rate of 40 mL/min. Subsequently, the temperature was raised to 500 °C, and the N_2_ was replaced with H_2_ to initiate the timer, with an H_2_ flow rate of 40 mL/min. Upon reaching the target treatment duration, the H_2_ was switched back to N_2_, and the temperature was lowered to obtain P@C-H_2_-x, where x represents the duration of H_2_ treatment in hours (x = 0, 2, 4, 6).

In addition, the catalytic activity of P@C was assessed under different H_2_ concentrations: designated as P@C(N_2_) under pure N_2_ carrier gas conditions and as P@C(N_2_ + H_2_) under the condition of 30 mL/min N_2_ and 10 mL/min H_2_.

### 3.2. Catalyst Characterization

X-ray diffraction (XRD) patterns were recorded with a MiniFlex 600 X-ray diffractometer (Rigaku, Tokyo, Japan), using filtered Cu Kα as a radiation source operating at 40 kV and 15 mA and operating with the step size of 0.02° and the scan rate of 3°/min in the 2θ range between 5–90°. Scanning electron microscopy (SEM, FEI Nova Nano 450, FEI, Los Angeles, CA, USA) was employed to examine the morphology of the samples. The specific surface area was determined using the Brunauer-Emmett-Teller (BET, Micromeritics ASAP 2460) method from the adsorption data. Temperature-programmed desorption of NH_3_ (NH_3_-TPD) experiments were performed using a Micromeritics Chemsorb 250 instrument (Norcross, GA, USA) equipped with thermal conductivity detectors (TCD). X-ray photoelectron spectra (XPS) were acquired using a Thermo Scientific K-Alpha^+^ photoelectron spectrometer (Waltham, MA, USA) with Al Kα radiation. ^31^P NMR characterization was conducted using an Agilent 600 M solid-state nuclear magnetic spectrometer (Santa Clara, CA, USA). Thermogravimetric analysis (TGA) data were collected using a TA SPT Q600 instrument (New Castle, DE, USA). 

Approximately 10 mg of the sample (the precise amount was measured) was loaded into an alumina crucible. Subsequently, the sample was heated to 800 °C at a rate of 10 °C/min under a flowing air atmosphere (100 mL/min), and the corresponding weight change was recorded throughout this process. Upon determining the carbon deposition, the carbon deposition rate (molCarbon/gCat./h) was calculated using the following formula:

(1) $$Carbon\;Deposition\;Rate=\frac{∆m}{M\times ∆t\times W}\;(molCarbon/gCat./h),$$

where ∆m is the amount of coke deposited on the catalyst (g), *M* is the molar mass of carbon (12.0 g/mol), ∆t is the reaction time (h), and *W* refers to the mass of the P@C catalyst (1.0 g).

### 3.3. Catalyst Tests

All experiments were conducted using a continuous flow fixed-bed stainless steel reactor (Tianjin Gold Eagle Technology Co., Ltd., Tianjin, China) fitted with a quartz tube liner measuring 850 mm in length and 10 mm in inner diameter. Precise control of the reaction temperature was achieved with an automatic temperature controller (Y-Feng Shanghai Co., Ltd., Shanghai, China), while the flow rate of *n*-heptane was meticulously regulated using a 2ZB-1L10 double plunger pump (Beijing Xingda Science & Technology Development Co., Ltd., Beijing, China). The carrier gas flow was precisely controlled using a mass flowmeter (Beijing Seven Star Co., Ltd., Beijing, China).

The typical evaluation conditions were as follows: 1.0 g of catalyst, at 500 °C, atmospheric pressure, a carrier gas flow rate of 40 mL/min, and an *n*-heptane model feed flow rate of 1.0 mL/h.

The products were analyzed via online gas chromatography (Agilent 7890B) employing a GS-GASPRO column (30 m × 0.32 mm). *n*-Heptane conversion as well as the product-selectivity were calculated based on the following equations:

(2) $$n\text{-}\mathrm{heptane}\;\mathrm{conversion}\%=\left(1-\frac{A_{\mathrm{heptane}}}{A_{\mathrm{total}}}\right)\;100,$$

(3) $$\mathrm{product}\;\mathrm{selectivity}\%=\frac{A_{i}}{\sum A_{i}}\;100\%,$$

where A_heptane_ was the *n*-heptane peak area, and A_total_ was the total peak area of both heptane and hydrocarbon products at the outlet. The selectivity was calculated by comparing the corresponding peak areas A_i_ to the sum of all product peak areas.

## 4. Conclusions

In this work, metal-free phosphorus-doped carbon catalysts were successfully prepared employing cost-effective and readily accessible renewable biomass carbon via a hydrothermal method and high-temperature carbonization procedure. Under reaction conditions of 500 °C and a WHSV of 0.68 h^−1^, P@C catalyzed the initial conversion of *n*-heptane at approximately 99.6%, with a toluene selectivity of 87.9%, demonstrating both high activity and selectivity for aromatic hydrocarbons. However, with increasing reaction time, the catalyst’s activity experienced a rapid decline, prompting further investigation into the potential causes of catalyst deactivation.

Although a small amount of coke was generated during the reaction, potentially covering the carbon surface and blocking pores, it was not identified as the primary cause of deactivation. Instead, thermal treatment in a reducing H_2_ atmosphere accelerated the deactivation rate of P@C. A longer hydrogen treatment time and higher hydrogen concentration were found to be associated with a swifter catalyst deactivation process, suggesting that hydrogen may mitigate the acidity of the catalyst and reduce the phosphorus-containing functional groups on its surface, thereby diminishing active sites. Therefore, timely removal of H_2_ from the system could prolong the lifespan of the P@C catalyst. In future work, we will conduct a more detailed and systematic exploration of its deactivation mechanism and further modifications will be made to improve its stability. This study provides valuable insights into the properties and design of metal-free carbon catalysts, with potential applications in the field of catalytic dehydroaromatization of hydrocarbons.

## Figures and Tables

**Table 1:** The performance of different catalysts for aromatization of *n*-heptane in the literature.

Catalyst	Reaction Condition ^1^	Con ^2^ (%)	S_Arom_ ^3^ (%)	Lifetime (h)	Ref.
Pt/KZSM-5(deAL)	550 °C, ambient pressure, WHSV = 2 h^−1^, H_2_/*n*-heptane = 6	96.1	B + T: 75.4	234	[11]
Pt/KBeta	550 °C, 0.1 MPa, WHSV = 2 h^−1^, H_2_/*n*-heptane = 6	80–100	70–80.7	160	[29]
Pt/Beta-Rb	550 °C, 0.1 MPa, WHSV = 2 h^−1^, H_2_/*n*-heptane = 6	78.5	T: 94.6	15.7	[9]
KPt@S-1	500 °C, 0.1 MPa, WHSV = 3 h^−1^, H_2_/*n*-heptane = 2	98	T: 62	180	[30]
6Pt/BaKL	420 °C, 0.1 MPa, WHSV = 0.68 h^−1^, H_2_/*n*-heptane = 6	97	T: 92	20	[14]
PtZn_3_/KL	500 °C, 0.1 MPa, WHSV = 0.68 h^−1,^ H_2_/*n*-heptane = 6	92	T: 86	25	[15]
Pt-Ce/γ-Al_2_O_3_	500 °C, 0.6 MPa, WHSV = 3 h^−1^, H_2_/*n*-heptane = 10	94.2	T: 29.2	/	[31]
Pt-5/KL	420 °C, ambient pressure, WHSV = 0.68 h^−1^, H_2_/*n*-heptane = 6	90	T: 89	27	[17]
PtFe-1/KL	420 °C, 0.1 MPa, WHSV = 0.68 h^−1^, H_2_/*n*-heptane = 6	90	T: 90	30	[16]
10Pt/KL	420 °C, 0.1 MPa, WHSV = 0.68 h^−1^, H_2_/*n*-heptane = 6	78 (2 h)	T: 82	20	[13]
P@C	500 °C, ambient pressure, WHSV = 0.68 h^−1^	99.6	T: 87.9	/	This Work

^1^ Weight hourly space velocity; H_2_/*n*-heptane (molar ratio); ^2^ *n*-heptane conversion; ^3^ aromatic-selectivity.

**Table 2:** Results of deconvolution of the P 2p and O 1s XPS peaks of the P@C after heat treatment in H_2_ atmosphere.

Sample	C (at %) *	O (at %)			P (at %)		
		C=O/P=O	C–O/P–O	H_2_O	C–O–P	C–P–O	C_3_–P
P@C	87.52	3.53	5.98	0.66	1.13	1.18	0.00
		34.7%	58.8%	6.5%	48.7%	51.3%	0.0%
P@C-H_2_-2	93.03	1.74	3.87	0.23	0.45	0.52	0.16
		29.8%	66.2%	4.0%	39.8%	46.3%	13.9%
P@C-H_2_-4	93.78	1.59	3.53	0.28	0.10	0.57	0.15
		29.4%	65.4%	5.2%	12.2%	69.5%	18.3%
P@C-H_2_-4-Used	94.26	1.25	3.37	0.30	0.07	0.43	0.32
		25.3%	68.5%	6.2%	8.6%	52.4%	39.0%

* at %: atomic ratio.

**Table 3:** Physicochemical properties of P@C-H_2_-x (x = 0, 2, 4, 6).

Sample	P (at %) ^1^	Total Acidity ^2^ (μmol g^−1^)	S_BET_ (m^2^ g^−1^)	Vt ^3^ (cm^3^ g^−1^)	D ^4^ (Å)	Carbon Deposition ^5^ (molCarbon/gCat./h)
P@C	2.31	137.60	1733.7	0.7876	5.50	1.20 × 10^−3^
P@C-H_2_-2	1.13	59.48	1699.8	0.7872	5.52	1.07 × 10^−3^
P@C-H_2_-4	0.82	25.83	1710.2	0.8014	5.54	0.60 × 10^−3^
P@C-H_2_-6	0.72	19.20	1723.8	0.8112	5.55	0.30 × 10^−3^

^1^ P atomic ratio obtained by XPS analysis; ^2^ Total acidity obtained by NH_3_-TPD analysis; ^3^ Volume calculated by the t-plot method; ^4^ Diameter calculated by Horvath-Kawazoe method; ^5^ Carbon deposition calculated by TG.

**Table 4:** Physicochemical properties of catalysts after reaction in different H_2_ concentrations.

Sample	Total Acidity ^1^ (μmol g^−1^)	S_BET_ (m^2^ g^−1^)	Vt ^2^ (cm^3^ g^−1^)	D ^3^ (Å)	Carbon Deposition ^4^ (molCarbon/gCat./h)
P@C(N_2_)-Used	29.56	946.3	0.4302	5.49	1.20 × 10^−3^
P@C(N_2_ + H_2_)-Used	28.54	1263.9	0.5759	5.40	0.81 × 10^−3^

^1^ Total acidity obtained by NH_3_-TPD analysis; ^2^ Volume calculated by the t-plot method; ^3^ Diameter calculated by Horvath-Kawazoe method; ^4^ Carbon deposition calculated by TG.

## Data Availability

The data that support the findings of this study are available upon reasonable request.

## Supplementary Materials

The following supporting information can be downloaded at: https://www.mdpi.com/article/10.3390/molecules29061288/s1, Figure S1: SEM image of the P@C; Figure S2: XRD pattern of the P@C and Pure C. Both samples show two typical diffraction peaks at 2θ of 24° and 44° which respectively corresponded to (002) plane and (100) plane; Figure S3: Comparison of products distribution (a) at 15 min and (b) at 260 min of P@C-H_2_-x; Figure S4: NH_3_-TPD curves of P@C-H_2_-x-Used (x = 0, 2, 4, 6); Figure S5: (a) N_2_ sorption isotherms and (b) pore size distribution of P@C-H_2_-x (x = 0, 2, 4, 6); Figure S6. NH_3_-TPD curves of P@C in different H_2_ concentrations; Figure S7: (a) N_2_ sorption isotherms and (b) pore size distribution of P@C in different H_2_ concentrations. Table S1. The product distribution of P@C-H_2_-x (x = 0, 2, 4, 6) after 15 min of *n*-heptane dehydroaromatization; Table S2. Acidity and textual properties of P@C-H_2_-x (x = 0, 2, 4, 6).
