# Supplementary material for: Exploring Deactivation Reasons of Biomass-Based Phosphorus-Doped Carbon as a Metal-Free Catalyst in the Catalytic Dehydroaromatization of n-Heptane
Source: Molecules. 2024 Mar 14;29(6):1288. doi: 10.3390/molecules29061288 (PMC10975870; doi:10.3390/molecules29061288)
Supplement: Supplementary file 1 [file molecules-29-01288-s001.zip › molecules-2904395-supplementary.pdf]

Exploring Deactivation Reasons of Biomass-Based Phosphorus-Doped Carbon as a Metal-Free Catalyst in the Catalytic Dehydroaromatization of *n*-heptane

Supplementary Information

Fei Yu [1,2], Siyuan Liu [1*] and Bo Liu [1]

[1] *Green Chemical Engineering Research Center, Shanghai Advanced Research Institute, Chinese Academy of Sciences, Shanghai 201210, China.*

[2] *University of Chinese Academy of Sciences, Beijing 100049, China*

liusiyuan@sari.ac.cn

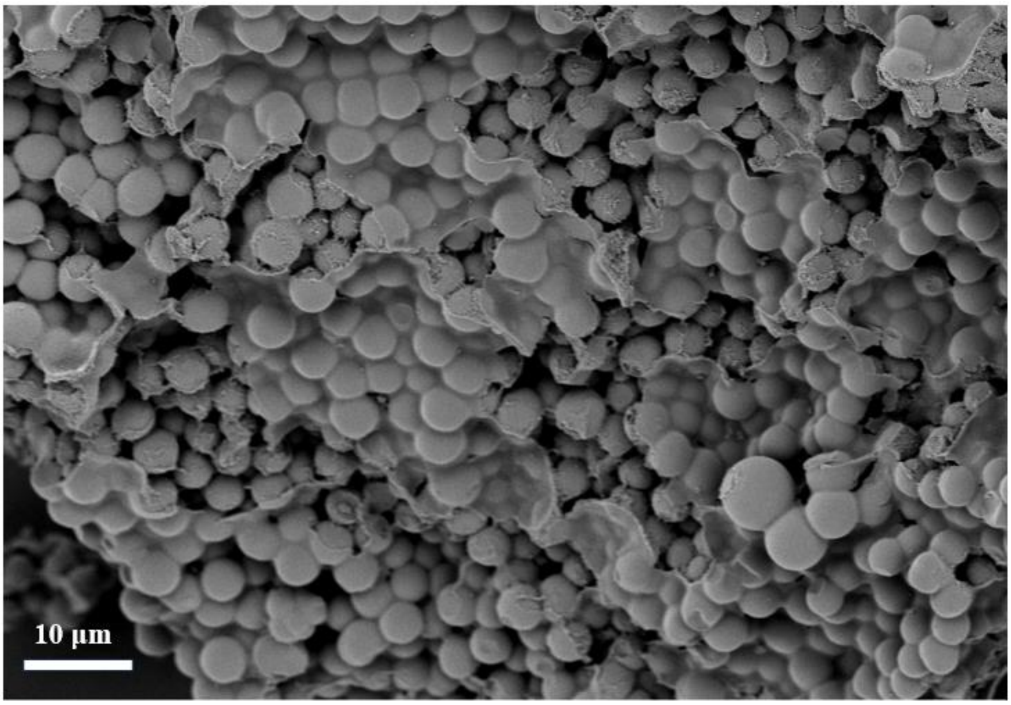

Figure S1. SEM image of the P@C.

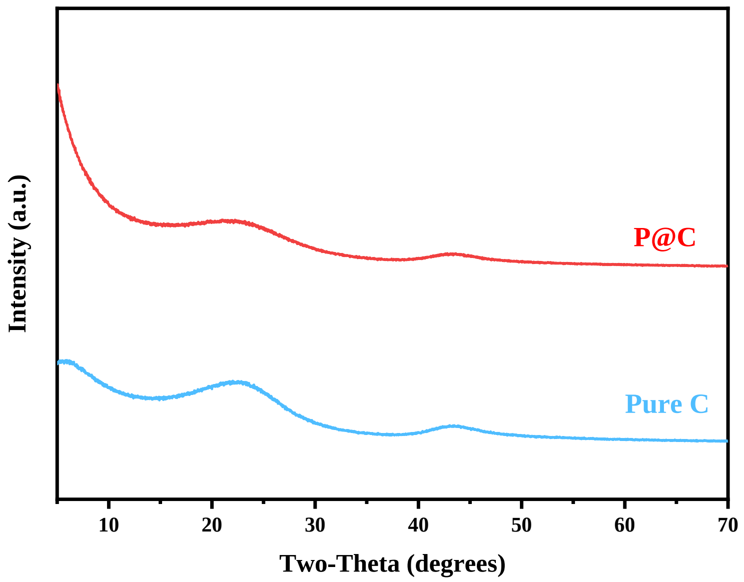

Figure S2. XRD pattern of the P@C and Pure C. Both samples show two typical diffraction peaks at 2θ of 24° and 44° which respectively corresponded to (002) plane and (100) plane.

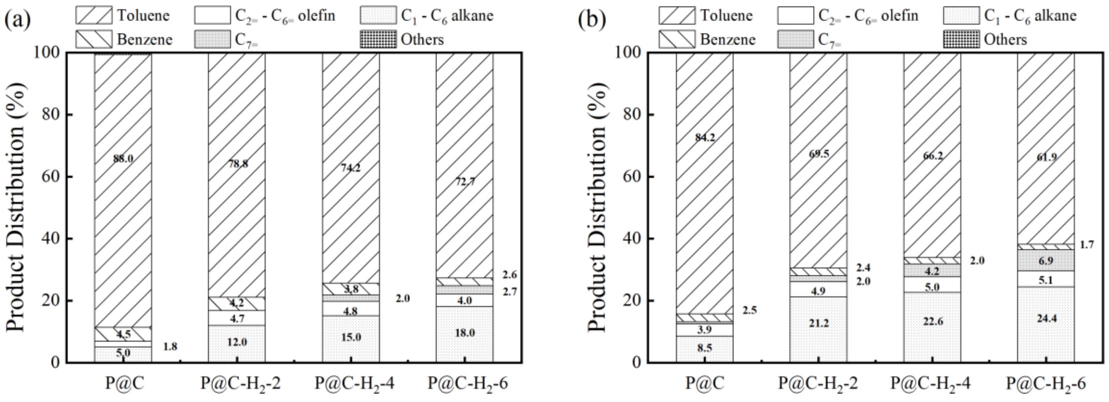

Figure S3. Comparison of products distribution (a) at 15 min and (b) at 260 min of P@C-H_2-x

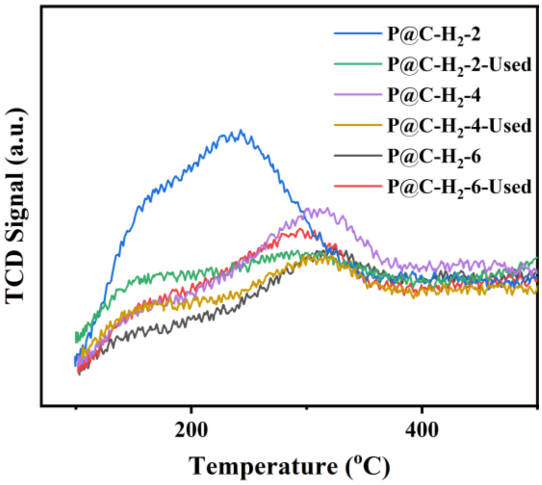

Figure S4. NH_3-TPD cruves of P@C-H_2-x-Used (x = 0, 2, 4, 6).

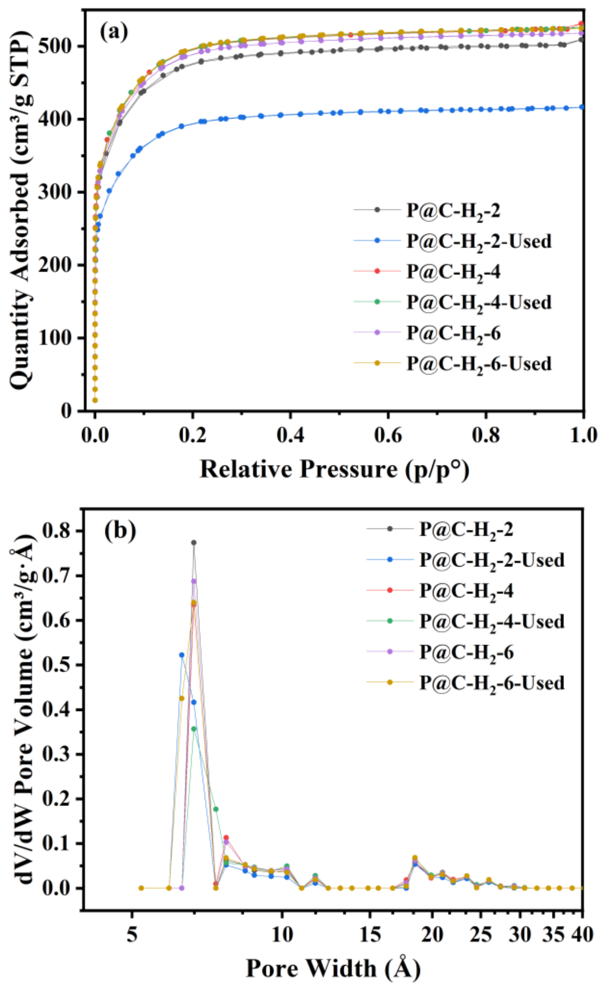

Figure S5. (a) N_2 sorption isotherms and (b) pore size distribution of P@C-H_2-x (x=0, 2, 4, 6)

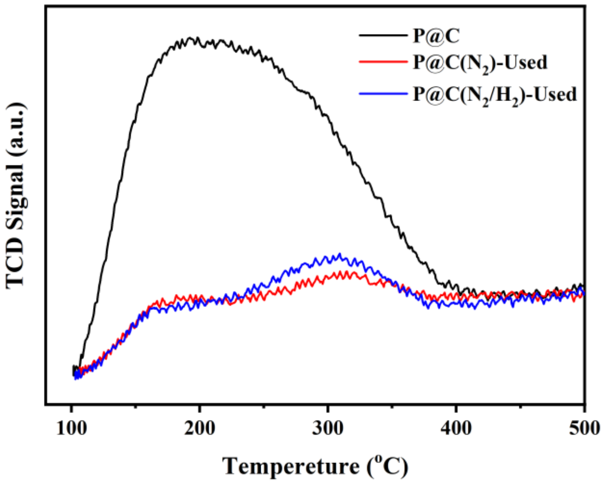

Figure S6. NH_3-TPD cruves of P@C in different H_2 concentration.

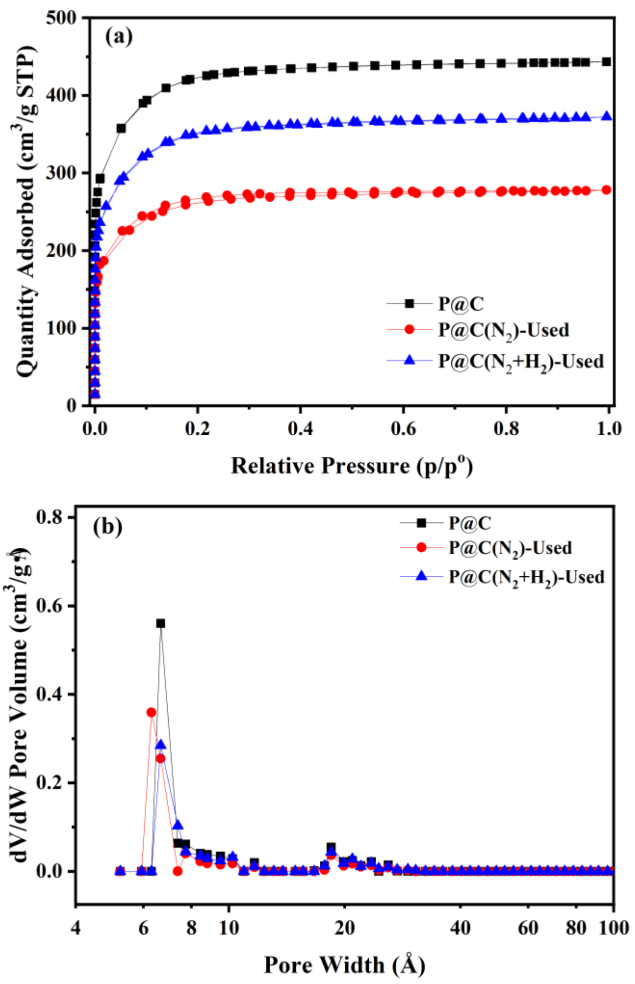

Figure S7. (a) N_2 sorption isotherms and (b) pore size distribution of P@C in different H_2 concentration

Table S1. The product distribution of P@C-H_2-x (x=0, 2, 4, 6) after 15 minutes of n-heptane dehydroaromatization.

Catalyst / Selectivity (%)	P@C	P@C-H_2-2	P@C-H_2-4	P@C-H_2-6
Toluene	87.93	78.80	74.21	72.70
Benzene	4.52	4.23	3.84	2.56
C1	1.61	1.14	1.32	1.30
C2	1.27	2.24	2.56	3.18
C2=	0.39	0.23	0.28	0.34
C3	0.92	3.43	3.97	4.62
C3=	0.67	0.90	0.87	0.78
C4	0.84	3.33	4.43	5.05
C4=	0.69	1.81	1.75	1.33
C5	0.38	1.64	2.24	3.19
C5=	0.07	1.22	1.30	1.17
C6	0.02	0.24	0.51	0.69
C6=	0.02	0.54	0.56	0.43
C7=	0.00	0.07	1.99	2.66
Other	0.67	0.18	0.17	0.00

Table S2. Acidity and textual properties of P@C-H_2-x (x = 0, 2, 4, 6).

**Sample**	**Total acidity** [1] **(μmol g^{-1})**	**S_{BET} (m^2 g^{-1})**	**Vt** [2] **(cm^3 g^{-1})**	**D** [3] **(Å)**
P@C-Used	29.56	946.3	0.4302	5.49
P@C-H_2-2-Used	21.69	1422.2	0.6443	5.48
P@C-H_2-4-Used	20.67	1515.5	0.7402	5.51
P@C-H_2-6-Used	17.94	1592.5	0.7614	5.52

[1] Total acidity obtained by NH_3-TPD analysis; [2] Volume calculated by the t-plot method; [3] Diameter calculated by Horvath-Kawazoe method; [4] Carbon deposition calculated by TG.
